# Carbon Redox-Polymer-Gel Hybrid Supercapacitors

**DOI:** 10.1038/srep22194

**Published:** 2016-02-26

**Authors:** A. Vlad, N. Singh, S. Melinte, J.-F. Gohy, P.M. Ajayan

**Affiliations:** 1 Institute of Information and Communication Technologies, Electronics and Applied Mathematics, Division of Electrical Engineering, Université catholique de Louvain, Louvain la Neuve, B-1348 Belgium; 2 Institute of Condensed Matter and Nanosciences, Division of Molecules, Solids and Reactivity, Université catholique de Louvain, Louvain la Neuve, B-1348 Belgium; 3 Institute of Condensed Matter and Nanosciences, Division of Bio- and Soft Matter, Université catholique de Louvain, Louvain la Neuve, B-1348 Belgium; 4 Department of Materials Science and Nanoengineering, Rice University, Houston, Texas 77005, United States

## Abstract

Energy storage devices that provide high specific power without compromising on specific energy are highly desirable for many electric-powered applications. Here, we demonstrate that polymer organic radical gel materials support fast bulk-redox charge storage, commensurate to surface double layer ion exchange at carbon electrodes. When integrated with a carbon-based electrical double layer capacitor, nearly ideal electrode properties such as high electrical and ionic conductivity, fast bulk redox and surface charge storage as well as excellent cycling stability are attained. Such hybrid carbon redox-polymer-gel electrodes support unprecedented discharge rate of 1,000C with 50% of the nominal capacity delivered in less than 2 seconds. Devices made with such electrodes hold the potential for battery-scale energy storage while attaining supercapacitor-like power performances.

Low energy density stored by the double layer of electrolyte ions at the surface of conducting carbon electrodes limits their practical implementation to specialty applications^1^,^2^,^3^,^4^. A major effort to increase the capacitance of electrical double-layer capacitors (EDLC) has been directed towards amplifying the specific surface area. Novel nanostructured carbons synthesis and assembly schemes led to the development of porous materials displaying specific surface in excess of 3100 m^2^/g^5^,^6^. Despite, the amount of stored energy is at best comparable to that of lead-acid batteries (in the range of 30 Wh/kg), whereas rather elaborated synthesis and device processing conditions are demanded and the intrinsic limitations of the carbon-based constituents seem to be attained^7^,^8^,^9^. One promising alternative for increasing the specific energy is the use of pseudo-capacitive components^3^,^10^. Such materials can be used solely or incorporated with EDLC carbon electrodes to further enhance the charge collection efficiency. Crucial prerequisites for efficient material hybridization are the proportional response of materials to the applied load (*i.e*., similar rate performances) but also simple integration schemes and easily available materials.

Since the electrochemical response of EDLC relies on very fast surface-ion exchange, only few materials comply^2^. Metal oxides such as RuO_2_ or Nb_2_O_5_, exhibiting near-surface or bulk insertion pseudocapacitive behavior, respectively, are representative examples^10^. Other metal oxides have been applied as well to increase the specific capacitance. Although nanostructuring can enhance ion insertion and extraction dynamics, sluggish reaction kinetics and phase transformation limit high power performances in such materials^3^,^11^. Electron conducting and redox biopolymers have been also extensively studied over the past years as potential candidates to increase the specific capacitance beyond that of double layer charge storage^12^. Nonetheless, these materials often suffer of poor electrolyte permeation (resulting in low ionic diffusivity and power limitations), cycling stability as well as low specific capacity and incomplete material utilization^13^,^14^. Hence, development of novel ultra-fast redox materials that moreover, are cheap to manufacture, easy to integrate and rely on abundant elements is continuously requested.

In this contribution, we demonstrate that organic polymer radical pseudocapacitors reveal ultra-fast, reversible, bulk-redox charge storage, commensurate to surface double layer ion exchange at carbon electrodes. Such attributes are assigned to the intrinsic electrical conductivity, very fast redox reaction kinetics and high ionic conductivity within the electrolyte-swollen polymer matrix that spans through the entire composite electrode. The incorporation of high surface area carbon shows a double benefit: EDLC charge storage and electrical doping for enhanced charge collection. With minimal manufacturing complexity, the hybrid electrode displays almost two-fold specific capacity and energy increase as compared to the pristine EDLC electrode, while being capable to maintain equivalent contribution of both components to the delivered charge, even at a load as high as 310 A/g. The versatility of the employed materials allows various devices fabrication schemes including symmetrical and Li-ion capacitors, the latter verging the energy density of Li-ion batteries. Such devices are safer than conventional inorganic batteries because they use non-flammable and transition metal free electrode materials, are adaptable to wet fabrication processes, easily disposable, flexible and can be fabricated via “green” chemical processes^15^,^16^,^17^.

The concept is schematized in Fig. 1. We used poly(2,2,6,6-tetramethyl-1-piperinidyloxy-4-yl methacrylate) (PTMA) organic radical redox material as a model pseudocapacitor system in our studies^18^. PTMA shows a reversible redox process at 3.6 V (*vs* Li/Li^+^) with a theoretical capacity of 110 mAh/g^19^,^20^. The hybrid electrode was constructed by incorporating PTMA within an EDLC electrode composed of high surface active carbon (~2,000 m^2^/g) and carbon nanotubes (Fig. 1A,B, see Materials and methods for fabrication details as well as Figure S1, Supporting Information for more structural and morphology details). Since these electrodes have been found to adopt a seamless carbon redox-polymer-gel morphology (see further) they are named C-RPG hereafter.

The electrochemical response of EDLC and C-RPG electrodes is shown in Fig. 1C. A typical linear voltage response was obtained for EDLC electrodes with a charge storage capacity of 38 mAh/g (voltage range 4.2–2.5 V *vs*. Li/Li^+^, 1 M LiPF_6_ in ethylene carbonate - diethylene carbonate electrolyte; corresponding to a specific capacitance of 80 F/g). Incorporation of PTMA induces a clear change in the electrochemical response. The C-RPG electrode displays a combined electrochemical response, with the Faradaic feature of the nitroxide (Fig. 1C, inset) being enclosed by the EDLC’s Coulombic response. The inclusion of the voltage plateau shifts the specific capacity of the C-RPG composite towards higher values, proportional to the amount of PTMA. At about 40 wt.% of PTMA, the specific capacity and energy density increases by as much as 70%: 38 to 63 mAh/g _total electrode_ and 127 to 220 Wh/kg _total electrode_ (total mass of electrode is considered here for both cases, Fig. 1D). Such significant improvements in the energy density provided by simple manufacturing seem very appealing.

To test the power performances and to determine the PTMA electrochemical response relative to fast surface ion exchange in EDLC, we have analyzed the rate response of C-RPG electrodes. The voltage profiles at various discharge rates are illustrated in Fig. 2A (see Figure S2 for symmetrical rate charge - discharge conditions). C-RPG electrodes show excellent capacity retention for rates of 1C to 100C (1C rate corresponding to a current density of ~63 mA/g). At higher rates, above 1,000C, the potential plateau of PTMA is shifted to lower potentials (see also Figure S3 for cyclic voltammetry results). Diffusion limitations and ohmic losses in the electrode as well as cell resistance could be accounted for this. Since the components are physically mixed, the charge collection at the PTMA/carbons interface could be affected. Covalent grafting^21^,^22^ or use of intrinsically conducting polymer redox materials^23^ could be envisioned to further address this issue.

Particularly interesting is the proportional contribution of both components and the presence of PTMA redox plateau even at high rates, in excess of 5,000C (Fig. 2A; see also Figure S4 for higher rate voltage-capacity profiles). Figure 2B shows the estimated specific contribution of both constituents to the discharge capacity function of C-rate. The capacity decays proportionally, with slightly better capacity retention for EDLCs. This hints to the fact that in the C-RPG electrodes the electrochemical response rate of PTMA is commensurate to the surface ion exchange of the carbon based EDLCs electrodes.

There are several particularities that endow C-RPG electrodes with such performances. The PTMA used here has a cross-linked macromolecular structure capable to swell considerable amounts of carbonate electrolyte adopting a polymer-gel morphology (Fig. 3A,C, Figure S5). Ionic conductivities as high as 2–5 mS cm^−1^ have been measured, comparable to those of standard gel-electrolytes (Fig. 3A)^24^. Upon swelling, PTMA inflates making a monolith polymer-gel phase with a seamless tri-dimensional interpenetrated carbon network (Fig. 3B, see also Figure S1). Therefore a fast ion transfer is reached within the PTMA and C-RPG layers. Fast reaction kinetics and no phase change in PTMA further support fast redox exchange at these electrodes^25^,^26^.

Next, we turn to the analysis of the charge collection efficiency. Despite the intuitive consideration that aliphatic chain polymers are electrically insulating, PTMA shows a non-negligible charge conduction of the order of 10^−2^ S cm^−1 ^17,27. The electrical conduction mechanism in PTMA is promoted by the concentration gradient driven charge transport^28^. In fact, intrinsic charge conduction was found so important that in an attempt to build a continuous PTMA-phase cell, using the PTMA as redox, gel-electrolyte and separator material, no relevant cycling was possible with fast self-discharge observed (see Figure S6 and related discussion). The addition of high surface area carbons and CNTs is found to enhance the charge collection, enabling orders of magnitude higher electrical conductivity, which is necessary for high power in C-RPG electrodes. Figure 3D shows the measured electrical conductivity of only carbon EDLC and C-RPG electrodes in both, dry and swollen states. High values of ~1.5 S cm^−1^ are retained after PTMA incorporation and electrolyte swelling as compared to pure carbon electrodes displaying electronic conductivities of 6 S cm^−1^.

The power performance of the C-RPG electrodes is consistent with the fast charge and mass transfer processes. In the designed C-RPG electrodes, each of the components has a precise and multitasking role: PTMA for pseudocapacitive energy storage and gel matrix for better electrolyte retention and safety yet, with limited diffusion constraints; high surface area carbon for surface double-layer charge storage and electrical conduction; whereas CNTs serve as dual mechanical and charge collection scaffold. Bulk ion transport, charge transfer and reaction kinetics are so fast that the charging is ultimately controlled by ohmic contributions (Figure S7), macroscopic diffusion as well as finite yet still elevated redox kinetics of the nitroxide radical^28^.

Having established the performances but also the limitations of the of C-RPG electrodes, we constructed two-electrode cells based on C-RPG material. The symmetrical C-RPG || C-RPG cell is discussed first. Figure 4A shows the voltage profile for the device cycled at a current density of ~60 mA/g. The nominal capacity of the cell is ~30 mAh/g based on the total mass of both electrodes (see Supporting Information for fabrication and characterization details). At an average cell voltage of 1.5V the stored energy is about 45 Wh/kg. The cell also shows stable operation, after 40,000 constant current charge and discharge cycles, 75% of its capacitance is retained with a Coulombic efficiency of 100 ± 0.5% *per* cycle. Cycling stability is also preserved at high temperature where the solubility of PTMA should be enhanced (Figure S8) and no such effect is observed here due to the highly cross-linked PTMA formulation^17^. Power performances of the symmetrical devices have been also determined and are discussed later (Fig. 4E).

The charge storage mechanism in the C-RPG symmetrical cell is different at both electrodes. At the positive electrode, the EDLC contribution from the high-surface area carbon additions to the capacity of PTMA whereas at the negative terminal, only EDLC contributes. To compensate the extra-capacity at the cathode, the anode polarization reaches values as low as 1.2 V (vs. Li/Li^+^, see Figure S9 and related discussion). The PTMA was found to be stable at these potentials (stability window 1.2–4.8 V *vs*. Li/Li^+^) enabling an additional safety element, that is terminal insensitive electrochemical device operation. The polarization reversibility is demonstrated in Fig. 4C. After 10 charge/discharge cycles (1^st^–10^th^ cycle) the polarity was inversed and cell was subjected to the similar cycling protocol (11^th^–20^th^ cycle). The full recovery of the cell performance was evidenced by re-inverting the polarity (21^st^–30^th^ cycle). Although such behavior is characteristic of symmetrical supercapacitors, the energy density of designed C-RPG || C-RPG cells is comparable to that of batteries, where terminal inversion could lead to irreversible degradation^29^.

To further increase the energy density, a prototype C-RPG lithium-ion capacitor (LIC)^30^ was assembled using a prelithiated graphite anode (cell configuration C-RPG || C_x_Li). The applied anode to cathode capacity ratio was 2 in order to balance the specific energy with the insertion kinetics at the graphite anode. With an average working voltage of 3.3 V, the designed C-RPG LIC delivers 40 mAh/g (based on the total mass of both electrodes) corresponding to specific energy of 130 Wh/kg (Fig. 4D). The theoretical amount of stored energy in such devices elevates at 170 Wh/kg (based on the mass of both electrodes in an optimally balanced cell configuration, see Supporting Information), that is potentially higher than that of lead-acid and NiMH cells (Fig. 4E). Even if considering low active material content in real cells, progressive values are attained, with power performances matching those of supercapacitors^31^.

Power and energy density characteristics for the tested devices are summarized in Fig. 4E. Since charge storage processes at both electrodes are fast, the symmetrical C-RPG cell shows power performances similar to that of supercapacitors. Yet, more energy can be stored as a direct consequence of PTMA incorporation into the C-RPG electrodes. A LIC, based on positive C-RPG and negative lithiated graphite electrodes can store similar amounts of charge but at much elevated voltage resulting in higher energy densities. In turn, power performances are affected, being limited by the de/insertion kinetics of graphite negative electrode (Fig. 4D). Lithiated hard or soft-carbon anodes could lead to enhanced power performances at the expense of lower energy due to higher working potential in such materials.

The results presented in this study establish that the broad class of organic radical pseudocapacitors^16^,^32^ have the potential of ultra-fast, reversible, bulk-redox charge storage, commensurate to surface double layer ion exchange at carbon electrodes. The hybrid C-RPG electrodes are shown to possess near-ideal electrode properties: high electrical conductivity and ionic diffusion, bulk redox properties with rapid redox kinetics with no phase change as well as stable cycling. The fact that such devices can deliver power densities similar to those of supercapacitors while containing energy amounts comparable to batteries, furthermore combined with simple processing of established materials and technologies, provides prospects for emerging energy storage technologies. While the true performance metrics of such devices will ultimately depend on the cell size or specific design^31^ the added performance enhancement value in carbon-based electrochemical capacitors is clearly demonstrated. The C-RPG promotes an entirely different type of supercapacitor electrode materials that could also find application as printed micro-supercapacitors or as hybrid technology energy storage cells^18^,^33^,^34^,^35^.

## Additional Information

**How to cite this article**: Vlad, A. *et al*. Carbon Redox-Polymer-Gel Hybrid Supercapacitors. *Sci. Rep*. **6**, 22194; doi: 10.1038/srep22194 (2016).

## Supplementary Material

Supplementary Information

## Figures and Tables

**Figure 1:**
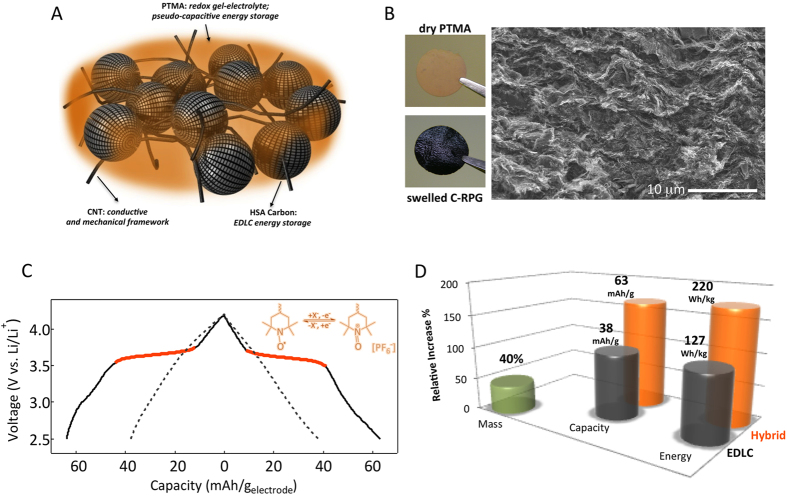
Hybrid carbon redox-polymer-gel (C-RPG) ultra-capacitor concept. (**A**) Schematic representation of the C-RPG electrode. The high surface-active carbon - CNT mat is flooded into a PTMA gel to generate the hybrid composite electrode. (**B**) SEM image of the C-RPG electrode. The layered morphology originates from the sequential deposition technique. Insets: optical micrographs of a dry PTMA pellet (top) and of a swelled C-RPG electrode (bottom). (**C**) Electrochemical response of the C-RPG and EDLC electrodes (charged and discharged at ~1C rate corresponding to 63 and 38 mA/g, respectively). The capacity is given per total mass of electrode. (**D**) Capacity and energy gain in C-RPG electrodes is as high as 170% upon incorporation of only 40% by weight PTMA.

**Figure 2:**
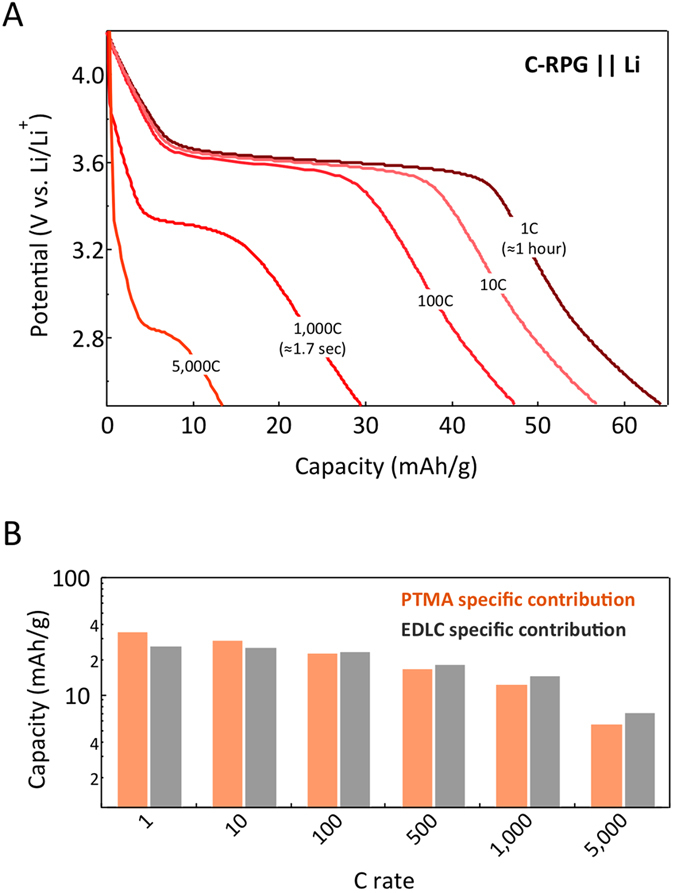
Ultra-high power performance. (**A**) Discharge profiles of C-RPG electrodes at C-rates ranging from 1 to 5,000. (**B**) Relative capacity contribution of PTMA and EDLC components function of the discharge rate.

**Figure 3:**
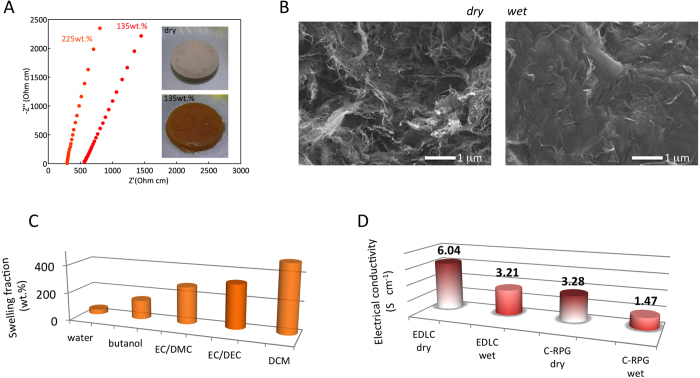
Hybrid electrode morphology and characteristics. (**A**) Electrical impedance spectra of the PTMA gels swollen with different amounts of liquid electrolyte (1 M LiPF_6_ in EC/DEC): 135 and 225 wt%. Inset: photographs of dry and electrolyte swelled PTMA pellets. (**B**) Morphology of the C-RPG electrode in the dry and electrolyte swollen state. In the gel form, C-RPG electrode adopts a seamless carbon – polymer gel configuration. Histograms of (**C**) liquids absorption of PTMA and (**D**) electrical conductivity of the EDLC *vs*. C-RPG electrodes in dry and electrolyte-swollen states.

**Figure 4:**
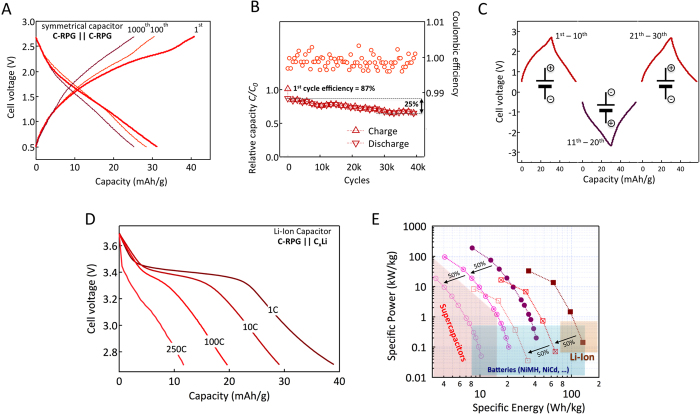
Symmetrical and lithium-ion hybrid capacitors. (**A**) Charge discharge profiles of a symmetrical C-RPG || C-RPG hybrid capacitor cycled at a rate of ~1C (corresponding to a current density of 60 mA/g _electrode_). (**B**) Capacity retention and Coulombic efficiency at a C-rate of 100. The first cycle efficiency is close to 90% with 75% of the capacity being subsequently retained after 40,000 cycles. (**C**) Terminal insensitive cell test. After inverting the polarization at the terminals, a similar response is obtained. (**D**) Discharge profiles of C-RPG || C_x_Li Li-ion capacitor at various C-rates. (**E**) Ragone plot for the prototype hybrid supercapacitors. Specific energy and power are also estimated considering only 50 and 25 wt.% active components content. For comparison, performance metrics for established energy storage devices are highlighted. Adapted from^36^.
