# Supplementary material for: Carbon Redox-Polymer-Gel Hybrid Supercapacitors
Source: Sci Rep. 2016 Feb 26;6:22194. doi: 10.1038/srep22194 (PMC4768163; doi:10.1038/srep22194)
Supplement: Supplementary Information [file srep22194-s1.pdf]

Supplementary Material for

Carbon Redox-Polymer-Gel Supercapacitors

A. Vlad[1]*, N. Singh[2], S. Melinte[1], J.-F. Gohy[3] and P.M. Ajayan[2]*

[1]Institute of Information and Communication Technologies, Electronics and Applied Mathematics, Electrical Engineering, Université catholique de Louvain, Louvain la Neuve, B-1348 Belgium. [2]Department of Mechanical Engineering and Materials Science, Rice University, Houston, Texas 77005, United States. [3]Institute of Condensed Matter and Nanosciences, Bio- and Soft Matter, Université catholique de Louvain, Louvain la Neuve, B-1348 Belgium.

Contact: alexandru.vlad@uclouvain.be, ajayan@rice.edu

Experimental Section

1. Synthesis of PTMA.

Cross-linked, carbon incorporated poly(2,2,6,6-tetramethyl-1-piperinidyloxy-4-yl methacrylate) (PTMA) was synthesized as previously reported[1,2]. In short, during the melt-polymerization of 2,2,6,6-tetramethyl-4-piperidyl methacrylate, 5 to 10% by weight carbon (Timcal Super C45 conductive carbon black, MTI Corp.) was added. The carbon enhances the electrical conductivity but here, was mainly used to ensure the brittleness of the composite and ensured fine milling of the PTMA powder (smaller than 100 nm polymer/carbon particles)[2].

2. Preparation and electrochemical characterization of separate components.

2.1 HiPco single walled carbon nanotubes (SWCNTs) were obtained from Rice University. The SWCNTs have been treated first with a mixture of concentrated HCl and H_2O_2 at 60°C for 12 h followed by abundant washing with water and drying[3]. The treated SWCNTs were the dispersed in n-butanol at a typical concentration of 50 to 100 mg/L. To determine the specific capacity of the SWCNT, a fabric was realized by vacuum filtration of approximately 2 mg/cm^2. The electrochemical characteristics are detailed in Table S1.

2.2 High surface active carbon (HSAC) was purchased from MTI and used as received (measured specific surface area > 1850 m^2/g). To determine the specific capacity of HSAC, a fabric containing 10% by wt. SWCNT was realized by vacuum filtration with a loading density of approximately 2 mg/cm^2. The electrochemical characteristics of HSAC were determined by substracting the SWCNT contribution as determined in 2.1; and are detailed in Table S1.

2.3 Timcal super C45 conductive carbon black was purchased from MTI and used as received. To determine the specific capacity of C45, a fabric containing 20% by wt. SWCNT was realized by vacuum filtration with a loading density of

[1] Vlad, A. *et al. Sci. Rep.* **2014**, *4*, 4315.
[2] Vlad, A. *et al. ChemSusChem* **2015**, 10.1002/cssc.201500246.
[3] Singh, N. *et al. Sci. Rep.* **2012**, *2*, 481.

approximately 2.5 mg/cm^2. The electrochemical characteristics of HSAC were determined by substracting the SWCNT contribution as determined in 2.1; and are detailed in Table S1.

2.4 To determine the specific capacity of PTMA (5 or 10 wt.% synthesis incorporated SC45), a fabric containing 20% by wt. SWCNT was realized by vacuum filtration with a loading density of approximately 2 mg/cm^2. The electrochemical characteristics of PTMA were determined by substracting the SWCNT and SC45 contribution as determined in 2.1; and are detailed in Table S1.

2.5 EIS conductivity of gelled PTMA were determined by first pressing PTMA pellet out of milled powders. Pellets with a thickness ranging from 100 to 200 um were analyzed. The pellets were gelled inside custom made Swagelok cells with Teflon rings spacers. The electrolyte was added drop by drop to allow full absorption within the PTMA matrix. The whole was then pressed between two stainless steel plates, sealed and EIS spectra acquired.

All electrode processing detailed in 2.1 - 2.4 were realized by first dispersing the constituents in n-butanol under sonication followed by filtration on a Nylon filter (Millipore).

3. Assembly of C-RPG electrodes and electrochemical characterization.

For the realization of C-RPG electrodes, several approaches were used, all providing similar results. Typically, a dispersion of PTMA(SC45) - HSAC - SWCNT in butanol was vacuum filtered to obtain free-standing electrodes (mass loading of approximately 2.5 mg/cm^2). The same dispersion was also successfully applied via sequential spraying on carbon-coated aluminum foil (pre-heated at 85 °C) at a lower mass loading, of approximately 1 mg/cm^2. Direct coating of butanol dispersion on aluminum foil failed as the film peeled and segregated during drying. To enable this, a C-RPG fabric was first hand grinded then ball-milled with CMC - SBR aqueous binder (3:2 weight ratio) at a total content of 5% by weight binder. The coating on aluminum foil was then performed at a mass loading of approximately 1.5 mg/cm^2.

The electrochemical characterization of separate constituents and C-RPG electrodes was first performed in a half-cell configuration using Li metal chip as reference and counter electrode (Alfa Aesar), one sheet of Celgard separator and 1M $LiPF_6$ in EC/DEC electrolyte (Novolyte) using CR2032 coin or Swagelock cells. The constant current charge - discharge measurements were performed in the potential range 2.5 - 4.2 V (*vs.* Li/Li^+) using Arbin BT-2043 multichannel potentiostat battery tester. EIS, cycling voltammetry and the fast data acquisition experiments (charge - discharge at rates above 1,000C) were realized using CHI660B potentiostat with a data acquisition rate of 2 ms.

Ultra high rate (above 500C) cycling tests were performed by pressing a small piece (mass of about 0.1 - 0.2 mg) of filtered C-RPG material into a stainless-steel mesh or by spraying equivalent amounts on carbon-coated aluminum foil.

The electrochemical performance of separate constituents and C-RPG electrodes are shown in Table S1. If otherwise stated, the capacity and energy values shown in the main text and SI are given per total mass of electrode (excluding the mass of current collector if used).

Table S1. Electrochemical and structural characteristics of individual components and of a typical C-RPG electrode.

Material	Specific capacity (mAh/g)*	C-RPG electrode composition**	
		mass loading (mg/cm^2)	weight %
PTMA[§]	98[#] (estimation error ± 4.5%)	1	40
HSAC	42[&] (estimation error ± 7%)	1.2	48.2
SWCNT	33[&] (estimation error ± 8%)	0.18	7.2
SC45	4 (estimation error ± 25%)	0.11	4.6
Total	---	2.5	100

Notes: * - Measured in the range 2.5 - 4.2 V *vs.* Li/Li^+, at a current density corresponding to 1C rate for each material. ** - Mass loading corresponding to a typical manufactured C-RPG electrode (for example, as shown in Figure 1C) using vacuum filtration. § - Containing 10% by weight SC45, the listed capacity excludes the contribution of SC45. # - Below 3 V, an extra capacity of about 5 - 10 mAh/g is gained[1] that is attributed to the second redox process in PTMA (nitroxide radical - aminoxyl anion)[4]. However, this process is kinetically unfavoured[3]. & - Consistent with previous reports.[5]

Table S2. Volumetric energy estimation.

Sample	Capacity (mAh)	Mass (mg)	Geometrical parameters			Specific capacity (mAh/cm^3)	Energy Density (Wh/L)
			thickness	area	volume		
#1	0.583	9.5	0.47 mm	31.25 mm^2	0.0147 cm^3	39.6	142
#2	0.128	2.1	0.12 mm	25 mm^2	0.003 cm^3	42.7	154

4. Assembly of EDLC carbon electrodes.

Only carbon EDLC electrodes have been also manufactured following the same protocol and with similar active material loading as C-RPG, of approximately 2 mg/cm^2. Only HSAC, SWCNT and SC45 were used in the same composition as shown in Table S1.

5. Symmetrical C-RPG capacitor assembly and testing.

Two identical C-RPG electrodes were sandwiched with one sheet of Celgard separator and soaked in the electrolyte. To gain in voltage, the negative electrode was first self-discharged to approximately 2 V (*vs.* Li/Li^+) via a series resistance of 10 - 50 kΩ. Due to partial self-discharge of this electrode, the OCP of assembled full cell was fluctuating in the 0.3 – 0.8 V range. The charge - discharge was performed in the 0.5 – 2.7 V voltage window.

6. Li-ion capacitor assembly and testing.

To assemble a Li-ion capacitor, the lithiation of the negative graphite electrode was first performed. The electrode was made by blending graphite (MTI), SC45 and PVDF in a 70 : 25 : 5 wt.% ratio with NMP and casting on Cu foil. The electrodes were dried at 55°C for 24 h and then transferred into an Ar-filed glove box for pre-lithiation. The pre-lithiation was performed either by simply short-circuiting a graphite/Li half cell for 30 - 45 minutes through a series resistance of 10 -

[4] Nishide, H. and Suga, T. *The Electrochemical Society Interface* **2005**, *Winter*, 32–36. Guo, W.et al. *Energy Environ. Sci.* **2012**, *5*, 5221.
[5] Wang, B. *et al., RSC Adv.* **2013**, *3*, 20024.

50 Ω or by controlled galvanostatic charge-discharge. The later was found to produce more accurate and reproducible results as typically 30% of the nominal lithiation capacity of graphite electrode (340 mAh/g reversible capacity obtained) was targeted. In this case, the electrode was first subjected to two slow discharge - charge cycles followed by partial discharge (at a rate of C/10). The potential profile is shown below:

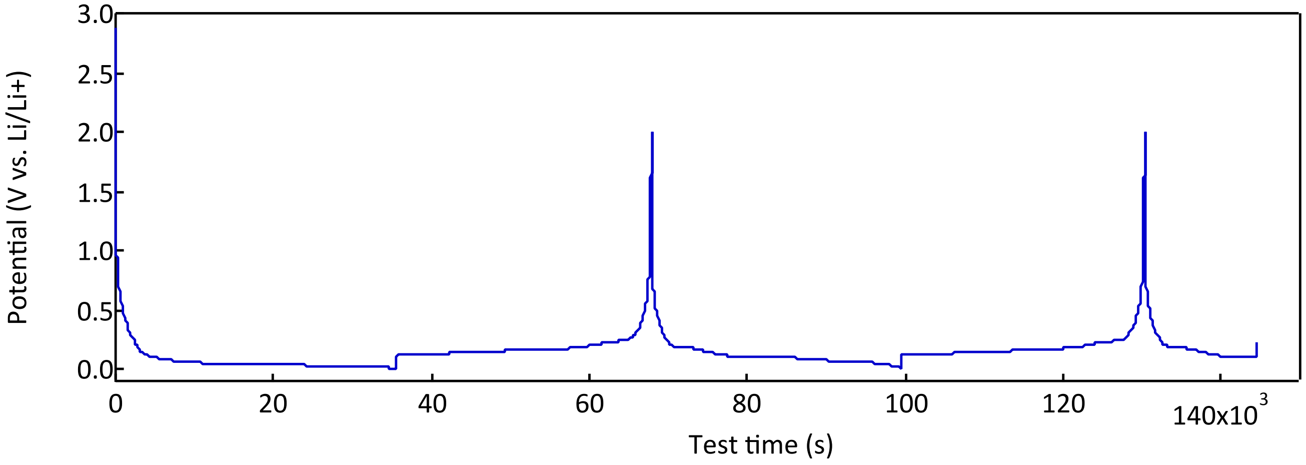

After, the cell was disassembled, the electrode rinsed with EC/DEC mixture and then assembled against a C-RPG electrode. One sheet of Celgard separator and 1M $LiPF_6$ EC/DEC electrolyte was used and the cycling window was limited to 2.7 – 3.7 V. The estimated cell balancing (positive to negative capacity ratio) was about 0.5 corresponding to mass ratio of approximately 3 ($\frac{P}{N} = \frac{2g\times 55mAh/g}{(0,85g\times 70\%)\times 340mAh/g}$). With optimized electrode composition, formulation and balancing (1:1 capacity ratio), a maximum theoretical capacity of $C = \frac{(6g\times 60mAh/g\ +1g\times 360mAh/g)/2}{6g+1g} = 51,5mAh/g$ could be reached. Soft or hard carbon negative electrodes can further improve the power performances of the used here graphite electrodes[6].

[6] Cao, W. *et al., Journal of The Electrochemical Society* **2014**, *161*, A2087–A2092.

Figure S1.

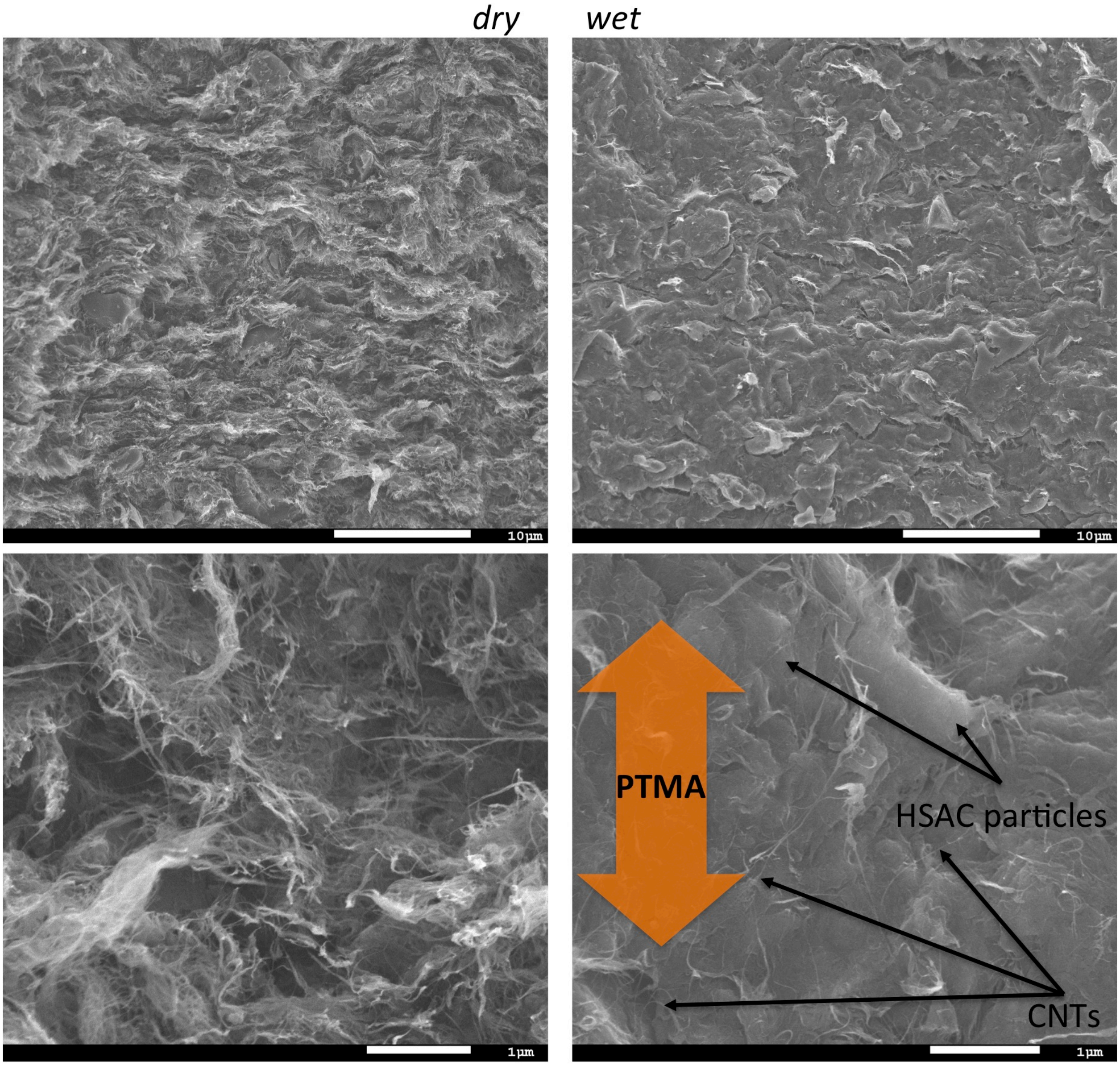

SEM of C-RPG before and after electrolyte impregnation. The PTMA monolith structure after impregnation ("wet") is generated because PTMA inflates in electrolyte. To generate these morphologies, C-RPG electrodes held between two pressed stainless steel plates were first dipped in 1M $LiPF_6$ in EC/DEC. The excess liquid was wiped and the whole let to dry to remove primarily the DEC component. The process was repeated several times until saturation was reached. The samples were then cleaved and the cross-section inspected with SEM. Thus, the observed morphologies could be assimilated to the operating gel-polymer morphology.

The composite has a polymer monolith morphology with carbon inclusions spanning through the entire structure. In fact, it can be regarded as a "bulk polymer" layer with carbon inclusions; the two types of carbons being

homogenously dispersed within the polymer phase (like schematically shown in Figure 1A, Main Text). At the microscale, the composite has a more heterogeneous structure as HSAC particles are in the micrometer-scale range and PTMA is not mixed (infiltrated) within HSAC. Carbon nanotubes (CNT) present are nevertheless uniformly spread and ensure the electronic conduction and mechanical support. The fact there is no intimate nanoscale mixing of AC with PTMA is not detrimental. In a control experiment (see also SI reference 2) PTMA was polymerized with HSAC and the electrochemical testing revealed lower power and energy performance than with the method described here. We believe this is due to the fact that the polymer is blocking the pores of HSAC (the surface area of thus fabricated HSAC-PTMA composite was lower). Thus, two detrimental effects are being assigned: first, the specific capacitance of HSAC is decreased and second, the power performances diminished.

In the C-RPG composite no such effect are present. The fact that the polymer is insoluble (highly cross-linked structure) inhibits the infiltration within the HSAC pores and leaves the access for electrolyte available. Thus, the full capacity and power performances of HSAC are recovered. And this is explained by the fact that electrolyte swelled PTMA acts like a gel-electrolyte with high ionic conductivity. At the same time, PTMA is heavily doped with conductive carbons (HSAC and CNT) and the electrical conduction is high. And given the fast redox kinetics of nitroxide radicals high power performances are attained. To recall, the main criteria for achieving high power (rate) performances are (i) high ionic conductivity; (ii) high charge conduction or low resistance and (iii) fast redox reaction kinetics. Thus, all the three requirements are meet in the C-RPG electrode explaining the attainable high rate performances.

Figure S2.

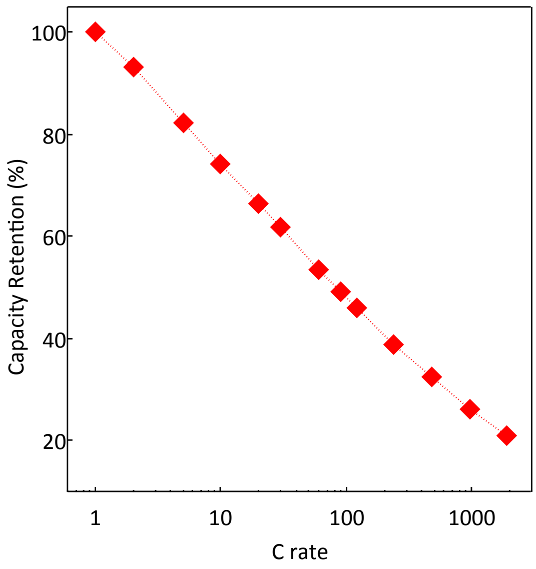

Normalized capacity retention of C-RPG electrodes under symmetrical cycling conditions (equal charge - discharge rates). Cycling voltage window : 2.5 – 4.5 V.

Figure S3.

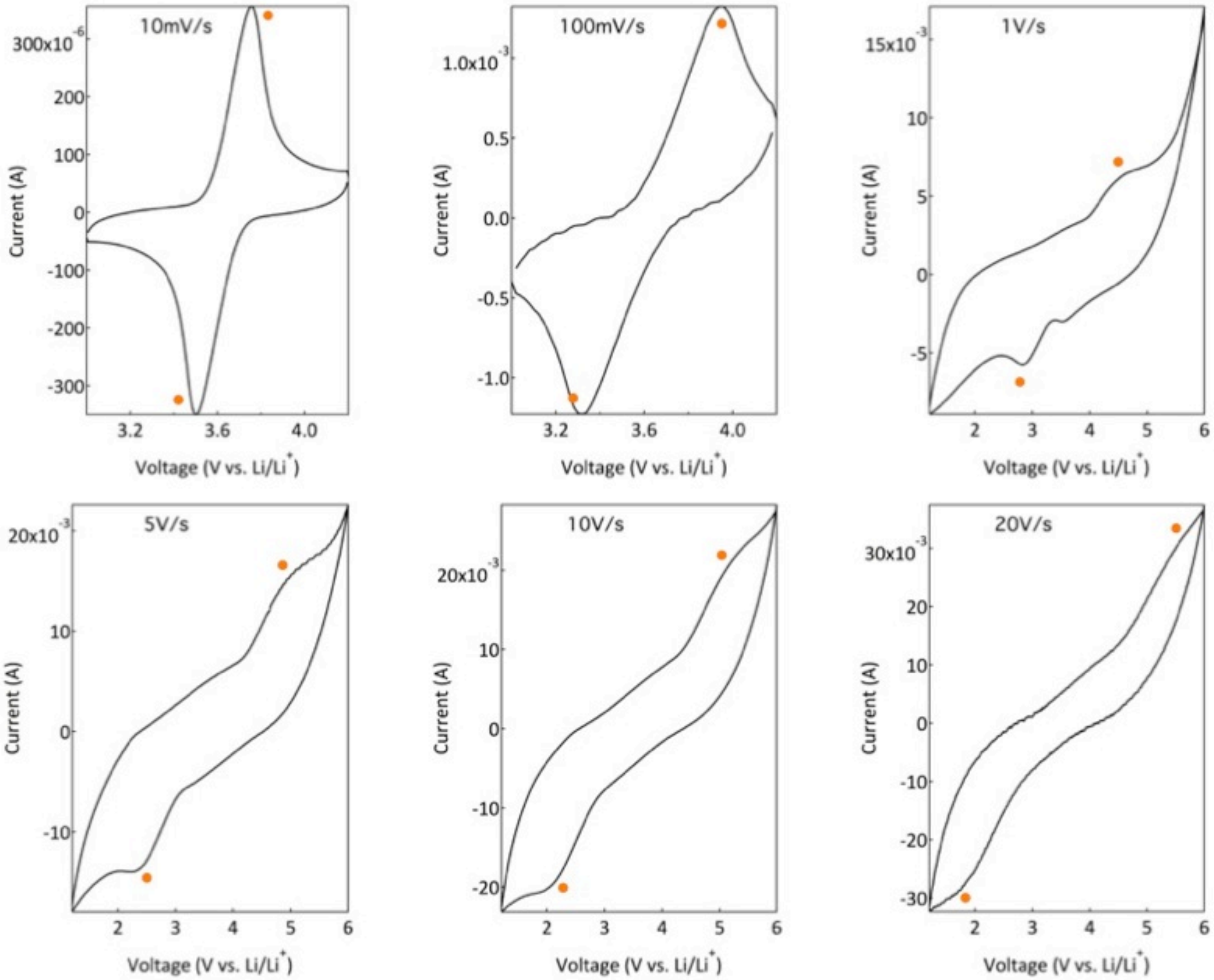

Cyclic Voltammograms of C-RPG electrodes recorded at scan rates ranging from 10 mV/s to 20 V/s demonstrating the high-rate capability of the C-RPG composite electrodes. The redox process of PTMA is marked with a dot.

Figure S4.

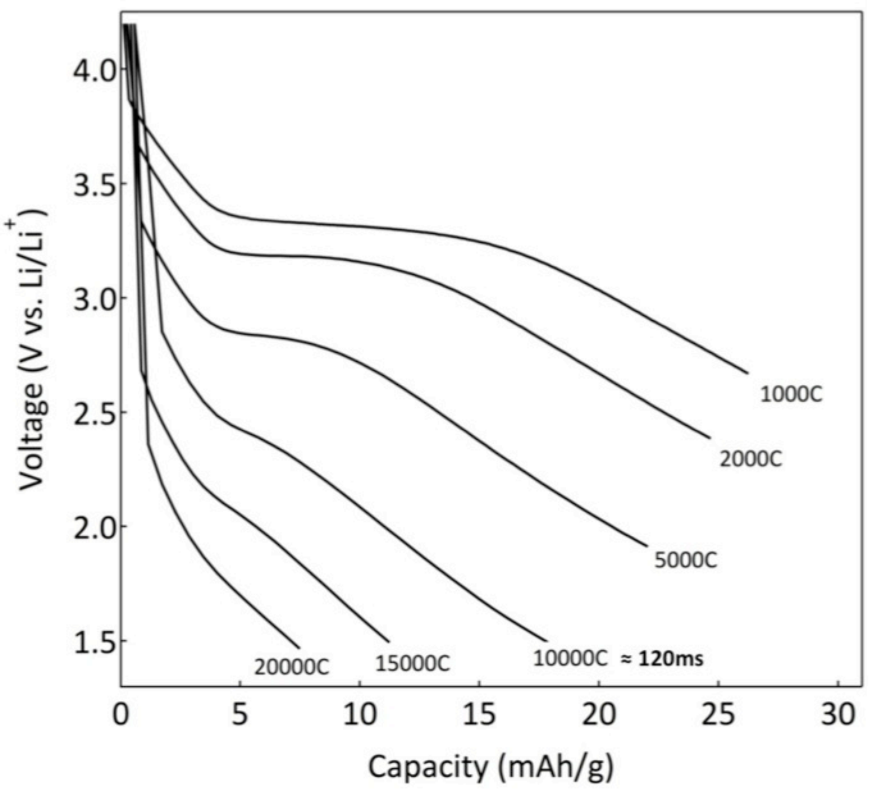

Ultra-high power performance of C-RPG electrode. The redox plateau of PTMA can be still visualized at a discharge rate of 15,000C. The ohmic voltage shift under high currents is important here (about 1 V at 10,000C). This could be improved by further optimization of charge collection and transfer efficiency. Since the composite electrodes are made by physical blending, we do not expect any chemical bonding between PTMA and the contained carbon. It should be mentioned that there is a strong correlation between the power performances and the charge resistance of the composite electrode as well as the interfacial resistance at the current collector. Corroborating previous studies,[7] we also found that the contact resistance at the current collector interface is crucial and the limiting step. But we also found that the rate (power) performances of the composite electrode strongly depend on the electron transfer resistance of the composite electrode. More studies are being required to get further insight and precisely determine the impact of these parameters on the power limitations.

[7] Yoshihara, S.; Isozumi, H.; Kasai, M.; Yonehara, H.; Ando, Y.; Oyaizu, K.; Nishide, H. *J Phys Chem B* **2010**, *114*, 8335–8340.

Figure S5.

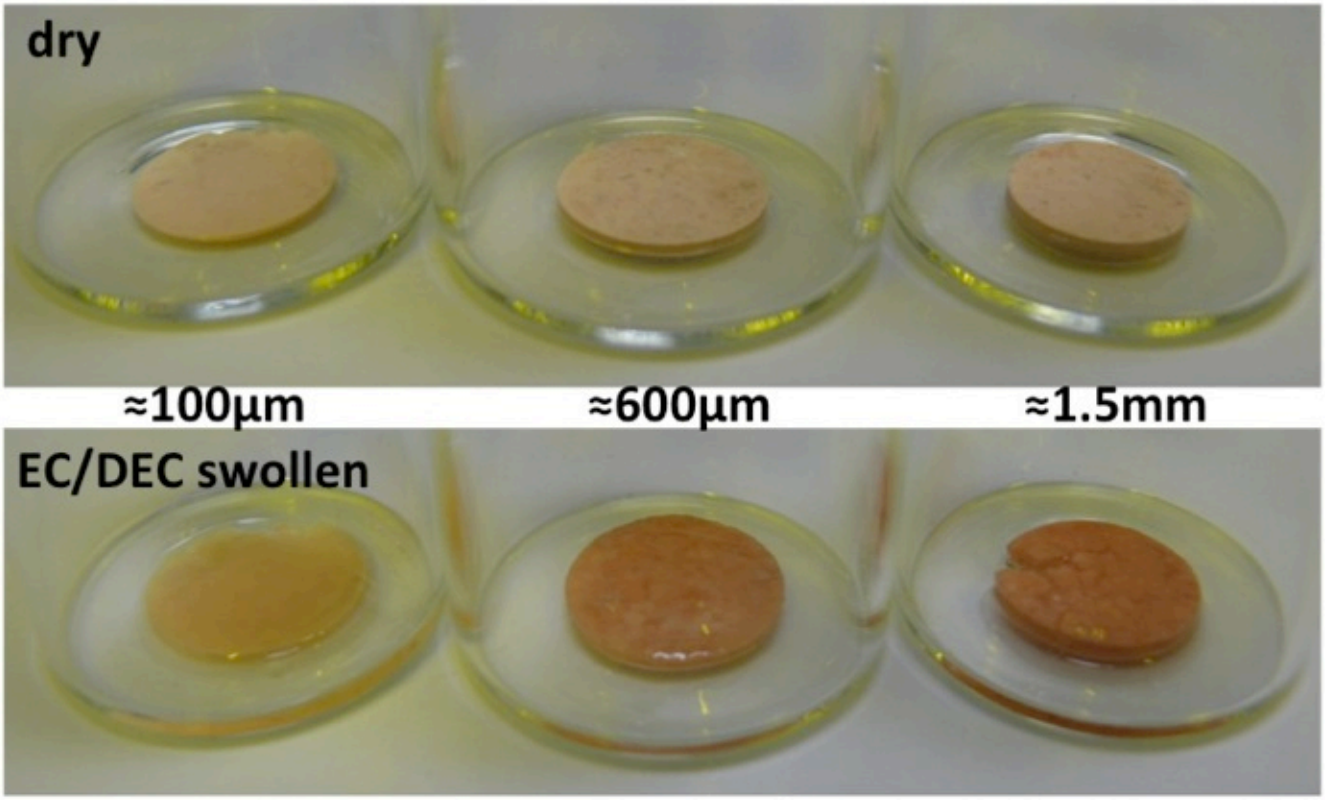

Optical micrographs of PTMA swelling process with 1M $LiPF_6$ in EC/DEC electrolyte. Thin PTMA pellets (< 600 μm thick) were found to maintain the structural integrity upon electrolyte uptake, whereas thicker samples where found to crack and disintegrate. CNT matrix in the C-RPG electrodes ensures mechanical reinforcement and effectively avoids the structural degradation.

Figure S6.

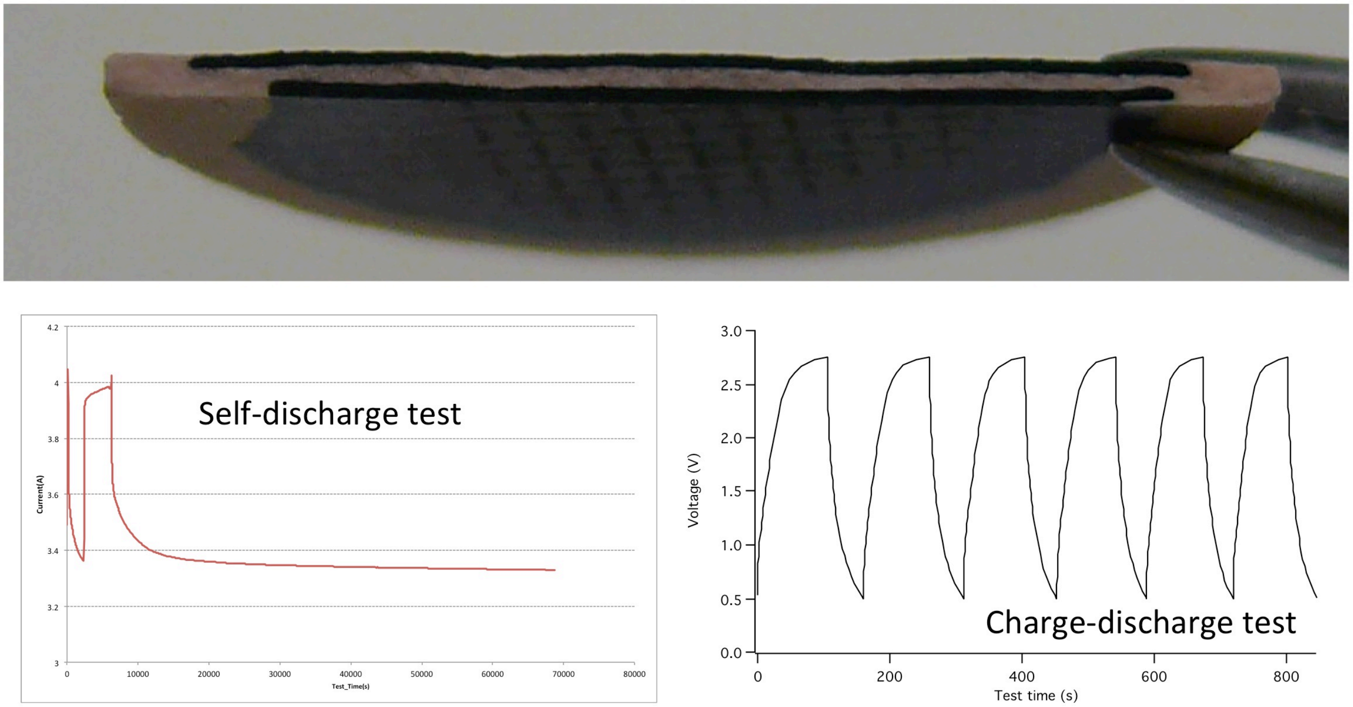

A continuous PTMA-phase hybrid symmetrical supercapacitor prototype was also build and tested. The device was made by pressing (@ 8Tonns) PTMA powder sandwiched between two C-RPG electrodes. The structure can be assigned to a continuous monolith PTMA phase. The whole structure was then swelled with electrolyte and electrochemical analysis performed. However, due to high charge conduction in the PTMA layer no relevant charging could be attained. The self-discharge test (C-RPG || PTMA || Li configuration) shows that the electrode shows no charge retention at 3.6V (equilibrium redox potential of PTMA) and the galvanostatic charge-discharge (C-RPG || PTMA || C-RPG configuration) displays poor efficiency and no redox features. Incorporation of charge propagation blocking barrier at the electrode - electrolyte interface could potentially avoid this.

Figure S7.

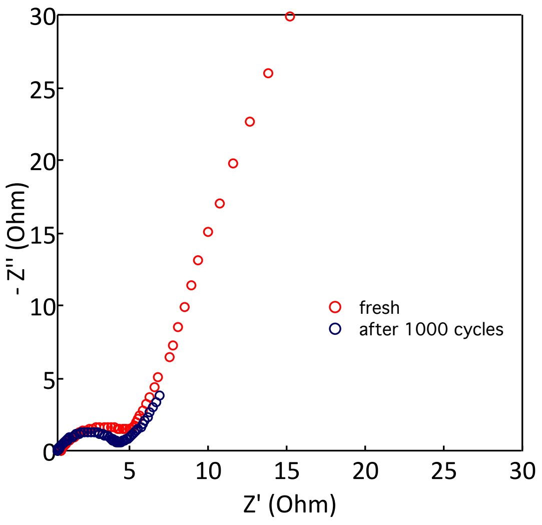

EIS of composite electrodes before and after 1000 charge-discharge cycles at a rate of 5C. Consistent with previous reports on PTMA-based hybrid batteries[1], an improved contact resistance and charge collection is noticed. This improvement could attributed to the amelioration of the PTMA - carbon and CNT interface during cycling. The oxidized PTMA has much higher affinity for the electrolyte (also noticed by higher solubility if not cross-linked) in the electrolyte and the repeated cycling renders much better interface with carbon. The migration of PF_6^- ions during cycling and associated chain swelling and rearrangement could also account for that.

Figure S8.

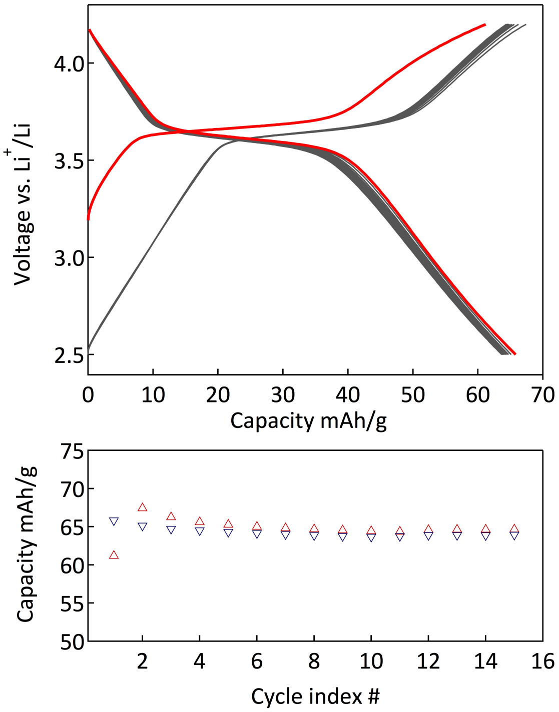

High temperature cycling (42°C, cycling rate C/10, 24 hours initial equilibration). Slow cycling was used so as to enhance the solubilization of PTMA, if any. No major degradation is observed.

Figure S9.

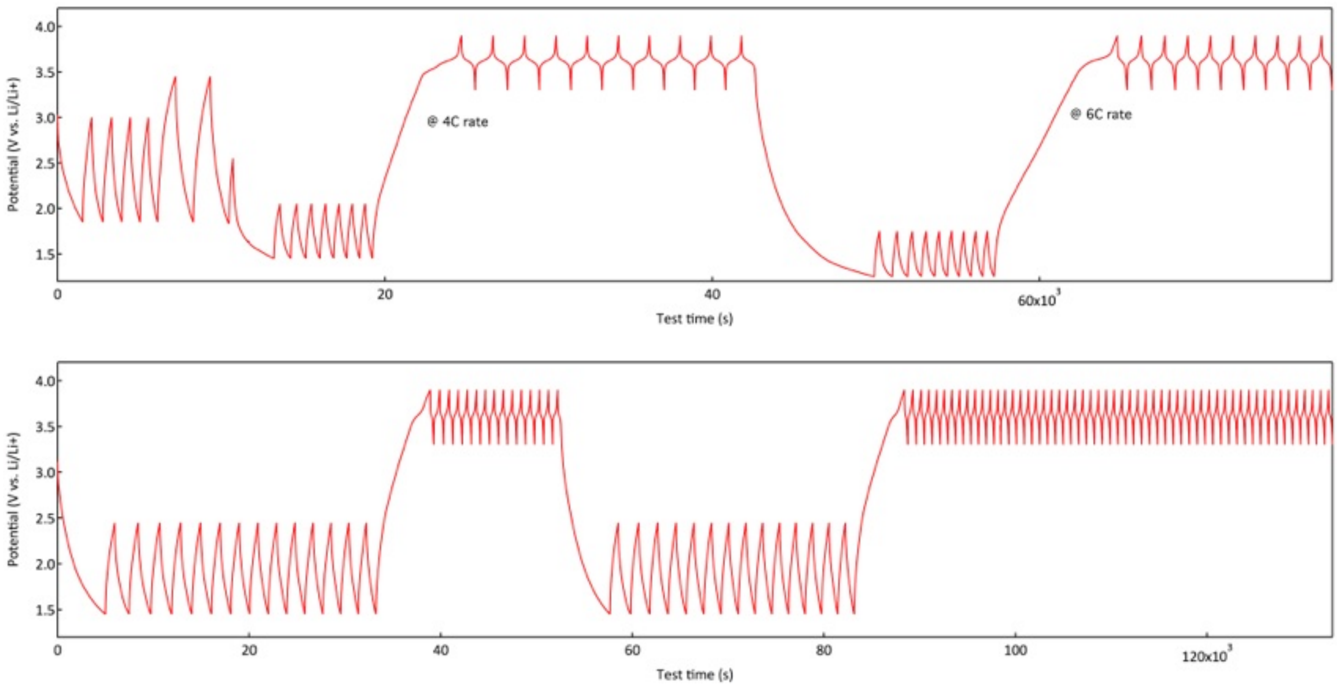

Typical charge-discharge curves of C-RPG/PTMA electrodes with different cut-off potential limits. Cycling between 3.3 - 3.9 V vs. Li/Li$^+$ was used to check the stability of the PTMA after being cycled at low potentials (2.5 - 1.5 V vs. Li/Li$^+$) where only EDLC charge storage contributes. Such measurements helped design and set the cycling limits for the symmetrical C-RPG capacitors. During their operation (as shown in Figure 4), at the positive terminal the redox of PTMA additions to the EDLC ion absorption at an average potential of 3.6 V (vs. Li/Li$^+$) whereas at the negative electrode, only ion exchange storage is involved at the high surface area of carbon at an average working potential of 2 V (vs. Li/Li$^+$).
